# Decreased interleukin‐17RA expression is associated with good prognosis in patients with colorectal cancer and inhibits tumor growth and vascularity in mice

**DOI:** 10.1002/cam4.7059

**Published:** 2024-03-16

**Authors:** Jeng‐Kai Jiang, Chi‐Hung Lin, Ting‐An Chang, Liang‐Chuan Lo, Chien‐Ping Lin, Ruey‐Hwa Lu, Chih‐Yung Yang

**Affiliations:** ^1^ School of Medicine National Yang Ming Chiao Tung University Taipei Taiwan; ^2^ Division of Colon and Rectal Surgery, Department of Surgery Taipei Veterans General Hospital Taipei Taiwan; ^3^ Institute of Microbiology and Immunology National Yang Ming Chiao Tung University Taipei Taiwan; ^4^ Department of Biological Science and Technology National Yang Ming Chiao Tung University Hsinchu Taiwan; ^5^ Cancer Progression Research Center National Yang Ming Chiao Tung University Taipei Taiwan; ^6^ Department of Pathology, Ren‐Ai Branch Taipei City Hospital Taipei Taiwan; ^7^ National Genomics Center for Clinical and Biotechnological Applications, Cancer and Immunology Research Center National Yang Ming Chiao Tung University Taipei Taiwan; ^8^ Department of Surgery, Zhongxing Branch Taipei City Hospital Taipei Taiwan; ^9^ Commission for General Education National United University Miaoli Taiwan; ^10^ General Education Center University of Taipei Taipei Taiwan; ^11^ Department of Education and Research Taipei City Hospital Taipei Taiwan

**Keywords:** colorectal cancer, interleukin‐17 receptor A, prognosis, tumor growth

## Abstract

**Background:**

Interleukin‐17 (IL‐17) is a pro‐inflammatory cytokine that plays a vital role in the promotion of tumorigenesis in various cancers, including colorectal cancer (CRC). Based on current evidence, IL‐17 binds to interleukin‐17 receptor A (IL‐17RA); however, the role of IL‐17RA has not been elucidated in previous studies on CRC. In this study, we explored the role of IL‐17RA in human CRC tissues and the progression of CRC in humans and mice.

**Methods:**

The expressions of IL‐17RA and epithelial‐mesenchymal transition (EMT)‐related genes were examined in CRC cells and tissue samples by quantitative real‐time polymerase chain reaction. The role of IL‐17RA in pathogenesis and prognosis was evaluated using a Chi‐squared test, Kaplan–Meier analysis, univariate, and multivariate Cox regression analysis in 133 CRC patients. A tumor‐bearing mice model was executed to evaluate the role of IL‐17RA in tumor growth, vascularity and population of infiltrating immune cells.

**Results:**

IL‐17RA expression was found to be significantly higher in CRC tissues than in adjacent normal tissues. The expression of IL‐17RA in Stage IV patients was significantly higher than that in Stages I and II patients. Patients with high IL‐17RA expression exhibited significantly worse overall and CRC‐specific survival than those with low IL‐17RA expression. Functional assessment suggested that the knockdown of IL‐17RA expression distinctly suppressed cellular proliferation, migration, invasion, and EMT‐related gene expression. In a tumor‐bearing mouse model, decreased IL‐17RA expression significantly repressed tumor growth and vascularity and reduced the population of regulatory T cells (Tregs) and myeloid‐derived suppressor cells (MDSCs).

**Conclusion:**

Reduced IL‐17RA expression also suppressed cellular proliferation, migration, and invasion, and the expression of EMT genes. Knockdown of IL‐17RA inhibited tumor growth and vascularity and decreased the population of Tregs and MDSCs in mouse tumors. Overall, IL‐17RA expression was identified to be independently associated with the prognosis of patients with CRC.

## 1 INTRODCTION

Colorectal cancer (CRC) ranks third among newly diagnosed cancers and is the third leading cause of cancer‐related deaths worldwide. More than 130,000 new CRC cases are diagnosed annually in the United States with approximately 1 million new patients and more than 500,000 deaths recorded every year worldwide.^1^ Despite initial tumor clearance due to effective cancer therapy, many patients with CRC develop recurrence and require treatment with chemotherapy and targeted agents. Approximately half of all patients with CRC develop Stage IV disease. According to the American Cancer Society statistics, the 5‐year survival rate reduces to 13% in patients with Stage IV disease.^2^ The high mortality in metastatic cancer is primarily due to drug resistance and molecular heterogeneity. Tumor resistance can be intrinsic or acquired during treatment, but can also occur via the positive selection of drug‐resistant subpopulations.^3^ A better understanding of CRC pathogenesis is of paramount clinical value to enhance the development of effective therapies. Early prediction of tumor progression can guide treatment strategies and improve patient outcomes. However, reliable biomarkers and simple prognostic tests for clinical practice are still required.

Interleukin‐17 (IL‐17), a pro‐inflammatory cytokine, promotes tumor initiation, growth, metastasis, and angiogenesis by transmitting its signal through IL‐17R.^4^, ^5^ IL‐17 alters the composition of tumor‐infiltrating leukocytes (TILs)^6^, ^7^ and reduces the sensitivity to chemotherapy^8^ and anti‐vascular endothelial growth factor (VEGF) therapy.^8^ Suppression of the IL‐17A‐IL‐17 receptor signaling pathway decreases the invasive capability of tumor cell lines in vitro.^4^, ^9^ In vivo, suppression of the IL‐17A pathway is associated with decreased tumor growth,^6^, ^8^, ^9^, ^10^, ^11^, ^12^, ^13^ increased tumor cell apoptosis,^6^, ^10^, ^11^, ^14^ decreased metastasis, and increased survival.^9^ The promotion of tumor development and progression owing to IL‐17RA engagement has been recognized for gastric cancer, non‐small cell lung cancer (NSCLC), and osteosarcoma.^4^, ^15^, ^16^ Interestingly, the association between clinicopathological features and IL‐17RA expression in CRC remains unclear. According to previous studies, negative and lower expression levels of IL‐17RA are significantly associated with advanced stage and poor prognosis in patients with CRC.^17^, ^18^ However, ablation of IL‐17RA expression was found to reduce tumorigenesis in a sporadic CRC mouse model.^10^ In our previous study, IL‐17A was highly expressed in serum and positively correlated with the number of circulating tumor cells, while negatively correlated with disease‐free survival in patients with CRC.^19^ Theoretically, IL‐17RA engagement enhances tumor proliferation, inflammatory responses, and lymphocyte infiltration, resulting in poor clinical prognosis.

In this study, we revealed that low IL‐17RA expression is associated with good prognosis and tumor progression. IL‐17RA knockdown was found to repress cellular proliferation, migration, invasion, and epithelial‐mesenchymal transition (EMT)‐related gene expression. Furthermore, decreased IL‐17RA expression inhibited tumor growth and modulated tumor‐infiltrating immune cells in mice.

## 2 MATERIALS AND METHODS

### 2.1 Colorectal cancer patient samples

Tumor and adjacent normal tissues were collected from 133 patients with CRC during surgical resection at the Taipei Veterans General Hospital. All study participants provided informed consent. The study was approved by the Institutional Review Board of Taipei Veterans General Hospital (VGHIRB number: 2016‐08‐008AC). Primary tumor staging was determined via histological examination. The patients were assessed for recurrence according to the following schedule: once every 3 months in the 2‐year period after surgical resection, once every 6 months in years 3–4, and annually thereafter. Chest radiography, abdominal sonography, and serum carcinoembryonic antigen (CEA) and carbohydrate antigen 19‐9 (CA19‐9) levels were used to assess recurrence. Abdominal/pelvic or chest computed tomography scans were performed annually. These scans were also performed to confirm recurrence when positive results were obtained.

### 2.2 Quantitative real‐time PCR

Total RNA was extracted using TRIzol Reagent (Life Technologies) following the manufacturer's instructions. For tissue samples, a homogenizer was used in addition to TRizol Reagent for tissue disruption. Reverse transcription was performed using either SuperScript III First‐Strand Synthesis System (Life Technologies) or RevertAid First Strand cDNA Synthesis Kit (Thermo Fisher Scientific). Gene expression was quantified via real‐time quantitative PCR using a SYBR Green PCR Kit and StepOne Real‐Time PCR System (Life Technologies). The raw data from four independent experiments underwent thorough analysis, with detailed results provided in Table S6. The following primer sequences were employed: human IL‐17RA, 5′‐ATGGACACTGCAGACAGACG‐3′ (forward) and 5′‐CTCACAGTCAGGCACAAGGA‐3′ (reverse); mouse IL‐17RA, 5′‐AGTGGACCCTGCAGACAGAT‐3′ (forward) and 5′‐CAGCATGGACAGAAACTGGA‐3′ (reverse); mouse E‐cadherin, 5′‐CCAAGCACGTATCAGGGTCA‐3′ (forward) and 5′‐ACTGCTGGTCAGGATCGTTG‐3′ (reverse); mouse N‐cadherin, 5′‐TTTCAAGGTGGACGAGGACG‐3′ (forward) and 5′‐CTCTCAAGTGAAACCGGGCT‐3′ (reverse); mouse Slug, 5′‐GCGAACTGGACACACACACACAGTTAT‐3′ (forward) and 5′‐CCCCAGTGTGAGTTCTAATGTGTCC‐3′ (reverse); mouse Vimentin, 5′‐AGCTAACCAACGACAAAGCC‐3′ (forward) and 5′‐ TCCACTTTGCGTTCAAGGTC‐3′ (reverse); human GAPDH, 5′‐TGGTATCGTGGAAGGACTCATGAC‐3′ (forward) and 5′‐ATGCCAGTGAGCTTCCCGTTCAGC‐3′ (reverse); and mouse GAPDH, 5′‐CAAGGAGTAAGAAACCCTGGACC‐3′ (forward) and 5′‐CGAGTTGGGAtAGGGCCTCT‐3′ (reverse).

### 2.3 Immunohistochemical staining and quantification

Paraffin‐embedded, formalin‐fixed tissue sections (3 μm thickness) were deparaffinized and rehydrated through a series of incubation in xylene, ethanol, and water. For antigen retrieval, the tissue sections were incubated in EnVision FLEX Target Retrieval Solution, Low pH (Agilent) for 30 min at 100°C in a pressure cooker. Endogenous peroxidase activity was blocked via incubation in 0.3% hydrogen peroxide in Tris‐buffered saline (TBS) for 30 min. Nonspecific binding was blocked via incubation in TBS with 0.1% Tween‐20 (TBST) and 5% bovine serum albumin (BSA) for 1 h. The tissue sections were then stained overnight with polyclonal rabbit antihuman IL‐17RA primary antibody (PAB27447, Abnova) in a moist chamber at 4°C. For visualization, the tissue sections were stained using the EnVision + Dual Link System‐HRP (DAB+) kit (Agilent Technologies), according to the manufacturer's instructions, before counterstaining with hematoxylin. All washing steps were performed with 0.05% Triton X‐100 in TBS. For angiogenesis quantification, a primary antibody against mouse CD31 (ab28364, Abcam) was used as an anti‐IL‐17RA antibody in the staining procedure.

To quantify IL‐17RA expression, staining intensity (I) was graded from 0 to 3. Definiens software (Aperio) was used to measure the distribution of staining (D) according to the percentage of each field of view (FOV) that stained positive. A composite IHC score was then calculated as DxI. Positive staining of the tumor cell region was calculated in the tumor tissue, and positive staining of the normal cell region was calculated in adjacent normal tissues. Each immunohistochemical (IHC) staining was scored by two experienced pathologists according to the staining intensity and distribution. To quantify CD31 expression, the threshold function in ImageJ was used to identify positively stained areas and calculate the area per FOV.

### 2.4 Wound healing assay

Control shGFP and shIL‐17RA CT26 cells were seeded at 3 × 10^4^ cells per well in two‐well culture inserts (Ibidi) for attachment overnight. The inserts were removed at time 0 and the cells were stimulated with 100 ng/mL recombinant murine IL‐17A (rmIL‐17A; R&D Systems). The channel formed by the insert was imaged at 0, 6, 9, 12, and 24 h. At each time point, the MRI wound‐healing tool in ImageJ was used to measure the channel area that lacked cells.

### 2.5 Cell migration and invasion assay

An in vitro cancer cell invasion assay was performed using 24‐well BioCoat Matrigel Invasion Chambers (BD Biosciences), according to the manufacturer's instructions. Membranes with 8‐μm pore size were used. A total of 5 × 10^4^ CT26 cells were added to the upper compartment of each well. The lower compartment was filled with 100 ng/mL of the standard cell culture medium with rmIL‐17A. The cells were allowed to invade for 24 h; thereafter, the chambers were fixed in 4% paraformaldehyde for 10 min. After removing the contents of the upper membrane surface, the number of invasive cells was determined by counting the 6‐diamidino‐2‐phenylindole (DAPI)‐stained cells from five randomly selected microscopic visual fields. Cell migration assays were performed according to the same procedure, except Millicell single well hanging inserts (Millipore) with 8 μm pore size in 12‐well plates were employed.

### 2.6 Western blot

Cells were lysed with RIPA solution supplemented with protease inhibitor cocktail (EMD–Millipore). Proteins were separated on SDS‐polyacrylamide gels and transferred onto PVDF membranes (EMDMillipore). After blocking with 5% BSA in TBS–0.1% Tween 20 (TBS‐T) at room temperature for 1 h, the membranes were incubated with anti E‐cadherin (#3195, Cell Signaling), anti‐N‐cadherin (#13116, Cell Signaling), anti‐Slug (#9585, Cell Signaling), anti‐Vimentin (#9585, Cell Signaling), anti‐IL‐17RA (E‐AB‐70147, Elabscience) and anti‐GAPDH (G8795, Sigma‐Aldrich) antibodies at 4°C overnight. The membranes were washed three times for 10 min each with TBS‐T and incubated with the appropriate horseradish peroxidase–conjugated secondary antibodies (Santa Cruz Biotechnology) for 1 h at room temperature. Western blot signals were visualized using SuperSignal West Femto Maximum Sensitivity Substrate (Thermo Fisher Scientific) and imaged using ImageQuant LAS‐4000 (GE Healthcare Life Science).

### 2.7 Lentivirus Transfection and CT26 Infection

To produce lentivirus for CT26 infection, HEK293T cells were seeded on a six‐well plate (7.5 × 10^5^ cells per well). On next day, HEK293T were transfected with 2.5 μg of lentiviral vectors containing 1.25 μg of targeted shRNA, 1.125 μg of pCMV‐deltaR8.91, and 0.125 μg of pMD.G (National RNAi Core Facility, Taipei, Taiwan) with T‐Pro NTR II transfection reagent (T‐Pro Biotechnology). After 24 h, the medium was substituted with DMEM containing 30% FBS. After 48 h, the supernatants were harvested. For infection, CT26 were seeded in a 24‐well plate (7.5 × 10^4^ cells per well) with the feeder‐free system and subsequently treated with virus (multiplicity of infection [MOI] = 10) for 24 h. On the next day, cells were selected by 8 μg/mL puromycin (P8833; Sigma‐Aldrich).

### 2.8 Cell culture

CT26 murine colon carcinoma cells were purchased from Bioresource Collection and Research Center (BCRC number: 60447) and grown in Dulbecco's modified Eagle's medium (DMEM) supplemented with 10% fetal bovine serum (FBS, Life Technologies) and penicillin–streptomycin‐amphotericin B solution. The cells were maintained in a humidified atmosphere at 37°C with 5% CO_2_. Plasmid constructs expressing short hairpin RNA (shRNA) targeting the mouse IL‐17RA gene were used to generate stable IL‐17RA knockdown cell lines, CT26 shIL‐17RA #2‐1 and CT26 shIL‐17RA #4‐1, via lentiviral transduction. The plasmid containing control shRNA targeting green fluorescent protein (shGFP, TRCN0000072200, target sequence: AGTACAACTACAACAGCCACA) or shRNAs targeting IL‐17RA (shIL‐17RA#2‐1, TRCN0000068057 target sequence: CCACAAATCCAAGATCATCTT and shIL‐17RA#4‐1, TRCN0000366616, target sequence: CTTCGGCACCTACGTTGTTTG) were also obtained from the National RNAi Core Facility (Taipei, Taiwan). The transduced cells were cultured in DMEM supplemented with 6 g/mL puromycin.

### 2.9 Animal model of colorectal cancer

Male BALB/c mice were purchased from the National Laboratory Animal Centre of Taiwan. At 6 weeks‐old, mice were subcutaneously administered 1 × 10^6^ control shGFP CT26 and shIL‐17RA CT26 cells in the left and right flanks, respectively. Tumor volumes were measured using the equation: $\text{Volume}=0.52\times \text{Length}\times {\text{Width}}^{2}$ at the indicated time. All animal study procedures were approved by the Ethics Committee of Animal Experimentation of the National Yang Ming Chiao Tung University.

### 2.10 Flow cytometry analysis of mouse tumors

Mouse subcutaneous tumors (0.5 cm diameter) were removed and placed inside gentle MACS C tubes (Miltenyi Biotec) with 1% FBS in phosphate‐buffered saline (PBS). Tissue dissociation was performed using the “Tumor Program” setting on a gentleMACS Dissociator (Miltenyi Biotec). Erythrocytes were removed using Red Blood Cell Lysis Solution (Miltenyi Biotec). Trypan blue was used to check the cell viability of the resulting single cell suspension. For flow cytometry analysis, 10^6^ cells were incubated with staining antibody for 30 min at 4°C. The following antibody combinations were used: FITC rat anti‐mouse CD31 antibody (561,813, BD) for endothelial cell staining; APC rat anti‐mouse CD8 (553,035, BD) and PE hamster anti‐mouse CD69 (553,237, BD) for activated T cells; PE rat anti‐mouse CD4 (557,308, BD) and APC rat anti‐mouse CD25 (557,192, BD) for regulatory T cells (Tregs); and PE‐Cy7 rat anti‐mouse CD45 (552,848, BD), PE rat anti‐mouse CD11b (557,397, BD) and FITC rat anti‐mouse Gr‐1 (551,460, BD) for myeloid derived suppressor cells (MDSCs). All antibodies were purchased from BD Biosciences. FACS data were analyzed using the Kaluza software (Beckman Coulter). Various flow cytometry analyses were conducted to characterize specific cell populations (Figure 4). CD31‐FITC staining was performed, and the count of positive cells reflects the CD31+ cell population. Additionally, CD8‐APC and CD69‐PE double antibody staining allowed for the statistical analysis of double‐positive values, representing the CD8+ CD69+ cell population. In the identification of CD45‐PE‐Cy7‐positive cell populations, gating was initially applied. Subsequently, within this CD45+ cell subset, double‐positive cells for Gr‐1‐FITC and CD11b‐PE were classified, delineating the CD45 + CD11b + Gr‐1+ cell population. Furthermore, CD25‐APC and CD4‐PE double antibody staining were employed, and the statistical analysis of double‐positive values denotes the CD25+ CD4+ cell population.

### 2.11 Statistical analyses

**FIGURE 1:**
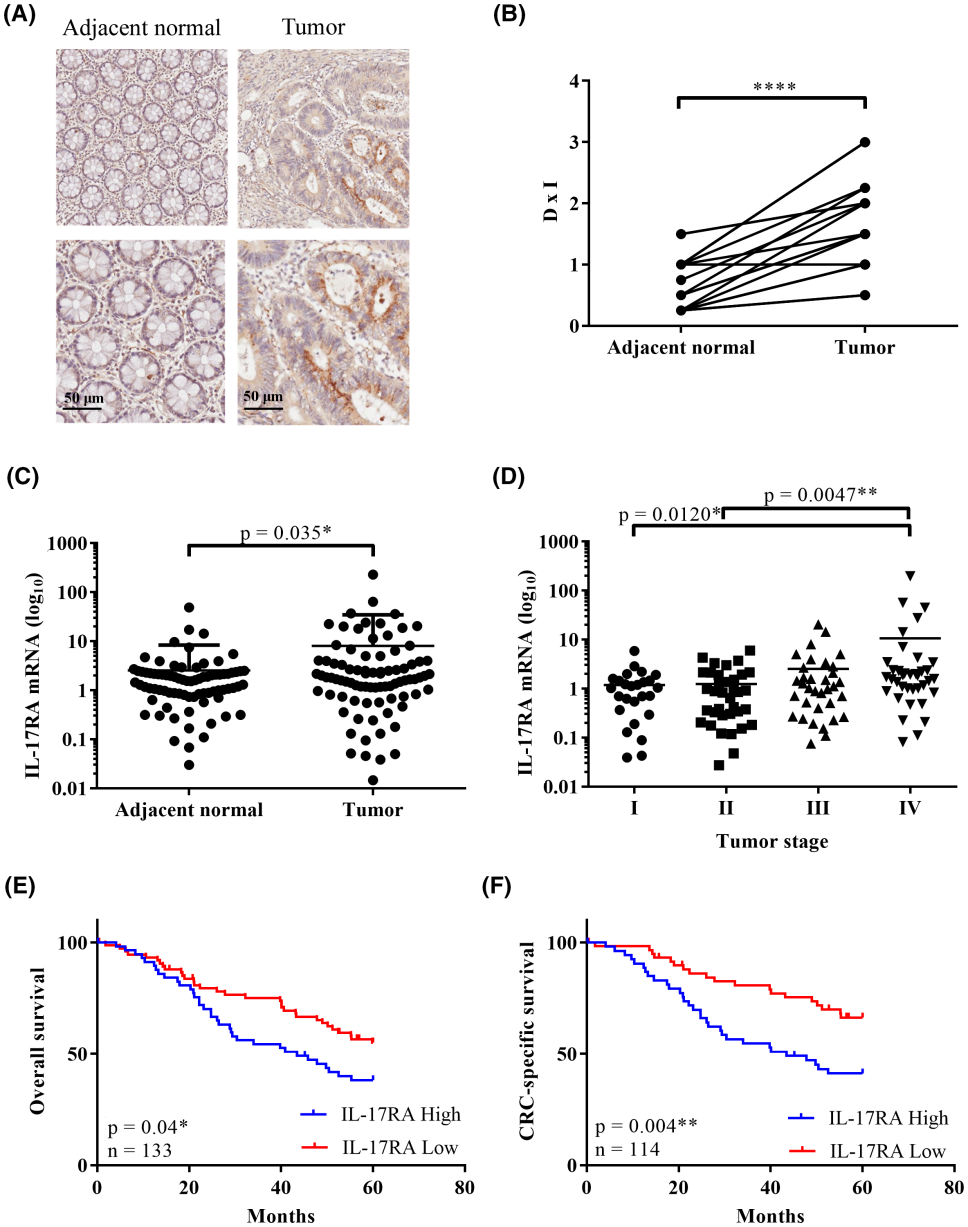
IL‐17RA expression in tumor tissues and prognosis in patients with colorectal cancer (CRC). (A) Immunohistochemical analysis of IL‐17RA distribution in paired CRC and adjacent normal tissue. Scale bar = 50 μm. (B) Expression level of IL‐17RA in the tumor region based on DxI, where D represents the percentage of IL‐17RA‐positive cells and I represents the immunohistochemistry staining intensity (****p < 0.0001, *N* = 20). (C) qRT‐PCR of IL‐17RA expression in paired tumor and adjacent normal tissue (*p = 0.035, *N* = 84). (D) qRT‐PCR of IL‐17RA expression in different stages (*N*: Stage I: 26, Stage II: 36, Stage III: 34, Stage IV: 37). (E) Kaplan–Meier analysis of overall survival according to IL‐17RA expression (*p = 0.040, *N* = 133). (F) Kaplan–Meier analysis of CRC‐specific survival according to IL‐17RA expression (**p = 0.004, *N* = 133).

**FIGURE 2:**
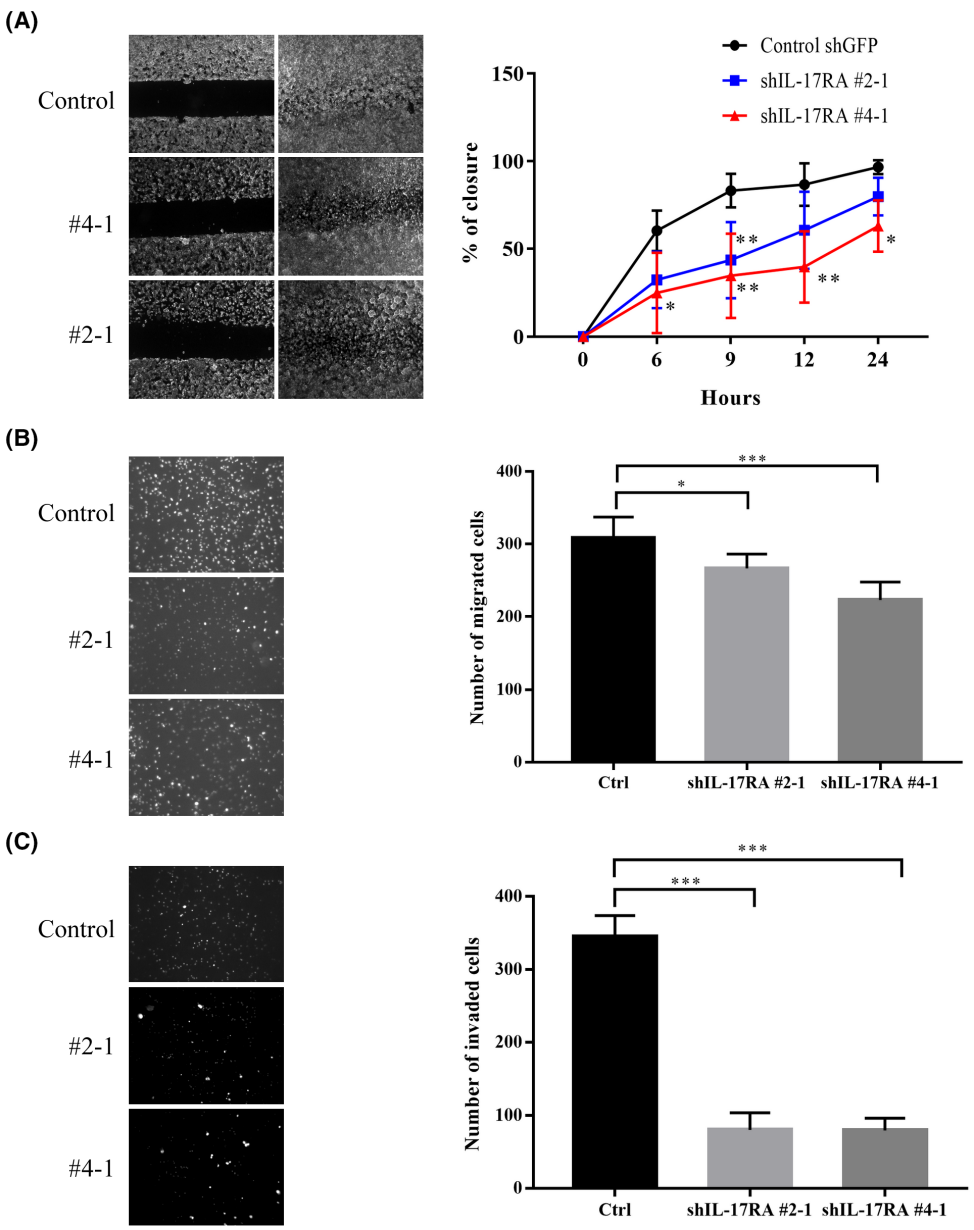
Knockdown of IL‐17RA expression inhibited tumor cell migration and invasion. (A) Wound healing assays of control shGFP and shIL‐17RA CT26 cells. (Left) Representative images of wounding at 0 and 24 h. (Right) Graphs of wound closure percentage from triplicate samples at 6, 9, 12, and 24 h. Mean ± SD for three independent experiments (*p < 0.05, **p < 0.01). (B) Transwell migration assay of control shGFP and shIL‐17RA CT26 cells. (Left) Representative images of migrated cells at 24 h. (Right) Graphs represent the mean number of cells in five regions of each well at random. Mean ± SD for five independent experiments (*p < 0.05, ***p < 0.001). (C) Invasion assay of control shGFP and shIL‐17RA CT26 cells. (Left) Representative images of the invaded cells at 24 h. (Right) Graphs represent the mean number of cells in five regions of each well at random. Mean ± SD for four independent experiments (***p < 0.001).

**FIGURE 3:**
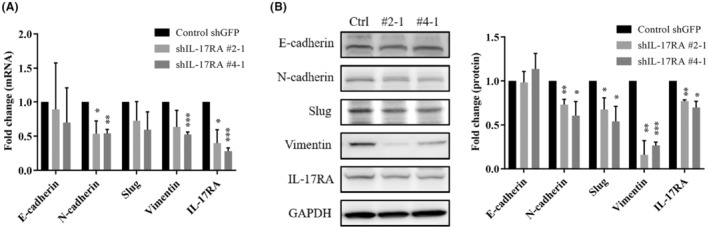
Knockdown of IL‐17RA expression suppresses mesenchymal‐epithelial transition‐related gene expression. (A) qRT‐PCR and (B) Western blot analysis of mesenchymal epithelial transition‐related mRNA and protein expression in shIL‐17RA and control shGFP CT26 cells Mean ± SD for four independent experiments (*p < 0.05, **p < 0.01, ***p < 0.001).

**FIGURE 4:**
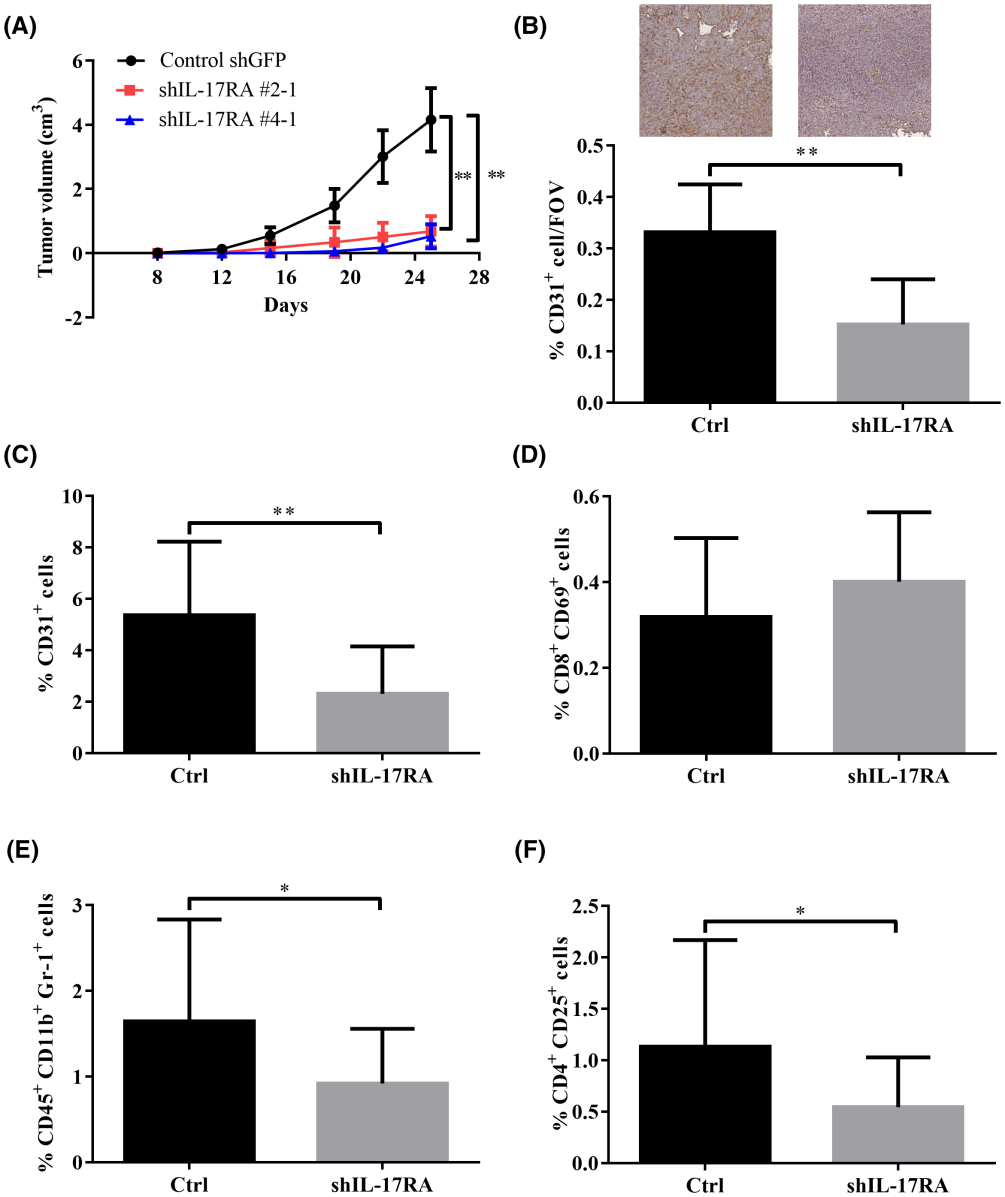
Knockdown of IL‐17RA expression significantly reduced tumor volume, angiogenesis, and percentage of regulatory T‐cells and myeloid‐derived suppressor cells (MDSCs) in a tumor‐bearing mouse model. (A) BALB/c mice bearing subcutaneous CT26‐control (shGFP) and IL‐17RA knockdown (shIL‐17RA) tumor. Tumor volumes are expressed as mean ± SD on the indicated days (Control shGFP, **p < 0.01, *n* = 10 mice per group; shIL‐17RA #2‐1, shIL‐17RA #4‐1, **p < 0.01, *n* = 5 mice per group). (B) IHC staining analysis for CD31 (*n* = 9 mice per group). Flow cytometry analysis of CD31^+^ cells (*n* = 10 mice per group) (C), activated T cells (*n* = 7 mice per group) (D), MDSCs (*n* = 9 mice per group) (E), and regulatory T cells (*n* = 10 mice per group) (F) in tumor lesions. The data were expressed as the mean ± SD *p < 0.05, **p < 0.01.

All data were statistically analyzed using GraphPad Prism (v6.0). The measurement data were expressed as mean ± standard deviation. The distributions of continuous variables are described as mean values and ranges. The Mann–Whitney *U*‐test and Wilcoxon signed‐rank test were performed to evaluate the differences between the groups and analysis of variance was used for comparisons among multiple groups, followed by Bonferroni corrections. *p*‐value <0.05 was indicative of a statistically significant difference. The analysis of effect sizes and corresponding confidence intervals was conducted for Figures 1, 2, 3, 4 and Table 1, with comprehensive details available in Tables S1–5. Survival was estimated using the Kaplan–Meier method and compared using the log‐rank test. Receiver operating characteristic (ROC) curve analysis was performed using GraphPad Prism (v6.0).

**TABLE 1:** Demographic and clinicopathological characteristics of patient cohorts segregated by patients with high or low IL‐17RA expression.

	No. of cases (%)	IL‐17RA high (%)	IL‐17RA low (%)	*p*
	*N* = 133	*N* = 58	*N* = 75	
Age				
≥65	74 (55.6)	39 (52.7)	35 (47.3)	0.0223*
<65	59 (44.4)	19 (32.2)	40 (67.8)	
Gender				
Male	83 (62.4)	36 (43.4)	47 (56.6)	>0.9999
Female	50 (37.6)	22 (44.0)	28 (56.0)	
TNM Stage				
I	26 (19.5)	8 (30.8)	18 (69.2)	0.0372*
II	36 (27.1)	12 (33.3)	24 (66.7)	
III	34 (25.6)	15 (44.1)	19 (55.9)	
IV	37 (27.8)	23 (62.2)	14 (37.8)	
T Stage				
T1–T2	31 (23.3)	7 (22.6)	24 (77.4)	0.0075**
T3–T4	102 (76.7)	51 (50.0)	51 (50.0)	
N Stage				
N0	75 (56.4)	25 (33.3)	50 (66.7)	0.0247*
N1	25 (18.8)	14 (56.0)	11 (44.0)	
N2	33 (24.8)	19 (57.6)	14 (42.4)	
M stage				
M0	96 (72.2)	35 (36.5)	61 (63.5)	0.0108*
M1	37 (27.8)	23 (62.2)	14 (37.8)	
Differentiation				
Poor	5 (3.8)	4 (80.0)	1 (20.0)	0.0259*
Moderate	124 (93.9)	54 (43.5)	70 (56.5)	
Well	3 (2.3)	0 (0.0)	3 (100)	
Location				
Right colon	46 (34.6)	19 (41.3)	27 (58.7)	0.8981
Left colon	57 (42.9)	25 (43.9)	32 (56.1)	
Rectal	30 (22.6)	14 (46.7)	16 (53.3)	
CEA (5 ng/mL)				
>5	56 (43.1)	31 (55.4)	25 (44.6)	0.0318*
≤5	74 (56.9)	26 (35.1)	48 (64.9)	
CA19‐9 (U/mL)				
>37	32 (26.0)	18 (56.3)	14 (43.8)	0.1504
≤37	91 (74.0)	37 (40.7)	54 (59.3)	
TIL				
Positive	30 (22.6)	14 (46.7)	16 (53.3)	0.8346
Negative	103 (77.4)	44 (42.7)	59 (57.3)	

*Note*: “*” indicates the *p*‐value range.

Abbreviations: CA19‐9, carbohydrate antigen 19–9; CEA, carcinoembryonic antigen.

* *p* < 0.05

** *p* < 0.01.

## 3 RESULTS

### 3.1 High IL‐17RA expression in tumor tissues is associated with poor clinical outcome in patients with colorectal cancer

To assess the expression levels of IL‐17RA in colorectal tumors, we examined the expression of IL‐17RA in human CRC and adjacent normal tissues using IHC staining (Figure 1A,B) and qRT‐PCR (Figure 1C). IL‐17RA expression in CRC tissues was significantly higher than that in adjacent normal tissues based on IHC and qRT‐PCR (Figure 1A–C). The qRT‐PCR results of different stages of CRC revealed that IL‐17RA expression was significantly higher in Stage IV than in Stages I and II (Figure 1D). High IL‐17RA expression was significantly associated with clinicopathological characteristics (Table 1). High IL‐17RA levels were significantly associated with old age, TMN classification, increased tumor invasion and metastasis, poor differentiation, and high CEA levels; however, no association with sex, tumor location, or CA19‐9 was found. Patients with high IL‐17RA expression had significantly worse overall survival (OS) (*p* = 0.04) and disease‐specific survival (*p* = 0.004) (Figure 1E,F). Cox regression analysis was conducted to validate the prognostic value of IL‐17RA in CRC‐specific survival. Univariate and multivariate analysis results, including IL‐17RA, age, clinical staging, CEA, CA‐199, and TILs, were analyzed to predict CRC‐specific survival (Table 2). The level of IL‐17RA is a potential predictor of CRC‐specific survival. Clinical staging and the levels of CEA and CA19‐9 were also identified as predictors of poor CRC‐specific survival. Multivariate analysis revealed that IL‐17RA was a predictor of CRC‐specific survival (HR = 2.221; CI = 1.135–4.345; *p* = 0.02) based on multiple logistic regression analysis after adjusting for age, sex, tumor stage, TILs, and CEA and CA19‐9 levels (Table 2).

**TABLE 2:** Univariate and multivariate analysis of colorectal cancer‐specific survival predictors by Cox regression model.

	Univariate cox regression			Multivariate cox regression		
	HR	95% CI	*p*‐Value	HR	95% CI	*p*‐Value
IL‐17RA (high versus low)	2.265	1.278–4.015	<0.0001****	2.221	1.135–4.345	0.020*
Age (≥ 65 versus < 65)	0.850	0.487–1.483	0.567	0.636	0.329–1.231	0.179
Stage (IV versus I–III)	3.499	1.996–6.133	<0.0001****	2.154	1.136–4.087	0.019*
CEA (>5 versus ≦5)	2.757	1.548–4.910	0.0006***	1.374	0.677–2.787	0.379
CA19‐9 (>37 versus ≦37)	3.635	2.022–6.533	<0.0001****	2.627	1.357–5.084	0.004**
TIL (positive versus negative)	1.218	0.636–2.331	0.552	1.569	0.762–3.321	0.221

Abbreviations: CA19‐9, carbohydrate antigen 19–9; CEA, carcinoembryonic antigen.

* *p* < 0.05; **p < 0.01; ***p < 0.001; ***p < 0.0001.

### 3.2 Knockdown of IL‐17RA expression decreases cellular migration and invasion

To elucidate the role of IL‐17RA in tumorigenesis, shRNA was employed to knockdown IL‐17RA expression in CT26 cells. First, we determined whether IL‐17RA plays a role in tumor migration and invasion. A wound healing assay was performed to elucidate the effect of IL‐17RA on tumor cell motility. The motility of IL‐17RA knockdown cells was found to be significantly decreased in the wound area (Figure 2A). Transwell migration (Figure 2B) and invasion (Figure 2C) assays also confirmed the significantly decreased migration and invasion of IL‐17RA knockdown cells. Therefore, knockdown of IL‐17RA had a significant suppressive effect on the migration and invasiveness of CT26 cells.

### 3.3 Knockdown of IL‐17RA expression leads to mesenchymal‐epithelial transition

EMT is believed to be an important process for the initiation of metastasis. We assessed the expression of EMT‐related genes and proteins in CT26 and IL‐17RA knockdown cells using qRT‐PCR and Western blotting. Based on the results, the expression levels of EMT‐related genes, including N‐cadherin and vimentin, were significantly decreased in IL‐17RA knockdown cells compared to those in control cells (Figure 3A). Furthermore, the expression levels of EMT‐related proteins, such as N‐cadherin, slug, and vimentin, were significantly lower in IL‐17RA knockdown cells compared to those in control cells (Figure 3B). Thus, the knockdown of IL‐17RA expression can suppress the expression of EMT‐related genes in CT26 cells.

### 3.4 IL‐17RA knockdown inhibits tumor growth and angiogenesis and impairs regulatory T cell and myeloid‐derived suppressor cell recruitment in vivo

A significant association was found between IL‐17RA expression and disease survival in patients with CRC (Figure 1E,F). To determine the role of IL‐17RA expression in tumor progression and pathogenesis, the growth rate, angiogenesis, and TILs in control and IL‐17RA knockdown CT26 tumor‐bearing mice were monitored. First, the growth rate was examined in IL‐17RA knockdown cells compared to control cells. Notably, IL‐17RA knockdown cells had a lower growth rate than control cells (Figure S1). In tumor‐bearing mice, mice bearing control cells had significantly larger tumor volumes than mice bearing IL‐17RA knockdown cells (Figure 4A; Figure S2). To assess tumor vascularity and the TIL population, IHC and flow cytometry were employed. Both IHC (Figure 4B) and flow cytometry (Figure 4C) revealed that IL‐17RA knockdown tumors had significantly decreased vascularity compared with control tumors. Cytometric analysis of the TIL population revealed significantly decreased MDSC (Figure 4E) and Treg (Figure 4F) populations in IL‐17RA knockdown tumors; however, no difference was found in the activated CD8^+^ T‐cell population (Figure 4D).

## 4 DISCUSSION

Although numerous studies have shown that IL‐17A and its downstream signaling contribute to poor prognosis in several cancers, including osteosarcoma, NSCLC gastric cancer, pancreatic carcinoma and CRC,^4^, ^10^, ^15^, ^16^, ^20^ the role of IL‐17RA in tumor development and progression remains unclear. In a mouse model, IL‐17RA engagement in colonic epithelial cells was found to induce the activation of p38 mitogen‐activated protein kinase (p38 MAPK), nuclear factor‐κB (NF‐κB), and extracellular signal‐regulated kinase (ERK) signaling molecules. This activation promotes cell proliferation and contributes to the transformation of enterocytes, leading to early tumor formation.^10^ IL‐17RA is overexpressed in several cancers, including gastric cancer, NSCLC, and osteosarcoma.^4^, ^15^, ^16^ High IL‐17RA expression is significantly associated with invasion of adjacent tissues, lymph node, and distant metastasis in gastric cancer, and poor OS in osteosarcoma, gastric cancer, and NSCLC.^4^, ^15^, ^16^ The overexpression of IL‐17RA can activate p38 MAPK in the NSCLC cell line.^15^ In NSCLC, the promotion of angiogenesis occurs through interleukin‐17, which stimulates the production of VEGF in cancer cells via the STAT3/GIV signaling pathway.^21^ In addition, the NF‐κB/ZEB1 signaling pathway mediates interleukin‐17‐induced EMT, fostering the migration and invasion.^22^ Furthermore, IL‐17RA involved in cell‐renewal of glioma cells.^23^ These studies imply that IL‐17RA may play a potential role in promoting tumor progression and cancer stem cell property. In our unpublished data indicates the IL‐17RA overexpression promotes cancer stem‐like properties of CRC cells by Stat3 activation.^24^ However, a study revealed that the deletion of IL‐17RA in CRC is linked to the degradation of A20, which is a negative regulator of the NF‐κB, WNT, and JNK‐c‐Jun signaling pathways, and associated with poor clinical outcome.^18^ Another study revealed that the low or loss of IL‐17RA is significantly associated with advanced stage tumors.^17^ In the present study, no abnormality was found in endogenous IL‐17RA expression in CRC tissues and adjacent normal tissues. Further, IL‐17RA expression was significantly higher in CRC tissues than in adjacent normal tissues based on RT‐qPCR and IHC (Figure 1A,B). High IL‐17RA expression was significantly associated with clinical stage, tumor invasion (T), lymph node metastasis (N), distal metastasis (M), differentiation, CEA, poor OS, and CRC‐specific survival. The IL‐17RA results for CRC are consistent with those for NSCLC, gastric cancer, and osteosarcoma, where high IL‐17RA expression was found to be associated with poor prognosis.^4^, ^15^, ^16^ Knockdown of IL‐17RA expression suppressed the expression of EMT genes, including N‐cadherin, slug, and vimentin, resulting in a decrease in cellular migration and invasion abilities (Figures 2 and 3). IL‐17RA expression was also higher in late‐stage tumors than in early‐stage tumors (Figure 1D). The univariate and multivariate analysis results, including Il‐17RA, age, tumor stage, CEA, CA19‐9 and TILs, were used to predict CRC‐specific survival. IL‐17RA has been identified as a predictor of poor survival. Based on multivariate analysis, IL‐17RA is a predictor of poor CRC‐specific survival based on multiple logistic regression analysis after adjusting for age, tumor stage, CEA, CA19‐9, and TILs (Table 2).

Cancer immune evasion is a major obstacle in designing effective anticancer therapeutic strategies.^25^ Tregs and MDSCs hamper effective anti‐tumor immune responses and are major players in tumor evasive mechanisms.^26^, ^27^, ^28^, ^29^, ^30^ In this study, the analysis of immune cell populations in mouse tumors revealed that the percentage of Tregs and MDSCs in IL‐17RA knockdown tumors was significantly lower than that in control tumors (Figure 4).^28^ Tregs and MDSCs have been demonstrated to represent predictors of the clinical outcome of patients with various cancers, including CRC.^31^, ^32^, ^33^, ^34^, ^35^ The infiltration of Tregs into tumors is linked to low survival rates and poor prognosis in various types of cancers, including colon, lung, ovarian, gastric, breast, and pancreatic cancers, as well as head and neck squamous cell carcinoma (HNSCC) and melanoma.^36^, ^37^, ^38^, ^39^, ^40^, ^41^ The increase in Tregs is commonly linked to the progression and metastasis of CRC, immunotherapy failure, and a poorer prognosis, although the correlation is not definitive.^42^, ^43^, ^44^, ^45^, ^46^ Conflicting evidence has thus complicated the picture. Surprisingly, conflicting evidence links a high density of Treg infiltration into CRC tissue with a favorable prognosis.^47^, ^48^, ^49^, ^50^ However, the role of tumor‐infiltrating Tregs in the development of CRC remains unclear. MDSC accumulation suppresses host immune responses and antitumor immunity, leading to tumor growth and progression in CRC.^51^, ^52^, ^53^, ^54^ The mechanism by which the IL‐17/IL‐17RA axis affects the recruitment of Tregs and MDSCs in tumors will be further explored.

In conclusion, the expression level of IL‐17RA is associated with the prognosis of patients with CRC. Decreased IL‐17RA expression impairs cellular proliferation, migration, and invasion, as well as EMT gene expression in CT26 cells. Furthermore, knockdown of IL‐17RA suppresses tumor vascularity and growth, and reduces the population of Tregs and MDSCs in mouse tumors. Since we found that high levels of IL‐17RA is negatively associated with CRC patient survival, besides, the population of Treg and MDSC cells was significantly lower in IL‐17RA‐knockdown tumor of mouse model. We can evaluate the relationship, mechanism and outcome prediction between IL‐17RA and Treg/MDSC in clinical CRC tissues in the future. In addition, we plan to explore the IL‐17/IL‐17RA pathway as a therapeutic target using humanized antibodies (secukinumab, ixekizumab, brodamulab) or small molecule inhibitors (S011806, LY3509754, LEO 153339) in mouse model and clinical trial.^55^, ^56^

## AUTHOR CONTRIBUTIONS

**Jeng‐Kai Jiang:** Conceptualization (lead); funding acquisition (equal); project administration (equal); resources (lead); supervision (equal). **Chi‐Hung Lin:** Conceptualization (equal); project administration (equal); resources (equal); supervision (equal). **Ting‐An Chang:** Data curation (equal); methodology (equal). **Liang‐Chuan Lo:** Data curation (equal); formal analysis (equal); methodology (equal). **Chien‐Ping Lin:** Data curation (equal); methodology (equal). **Ruey‐Hwa Lu:** Methodology (equal). **Chih‐Yung Yang:** Conceptualization (equal); data curation (equal); funding acquisition (equal); project administration (lead); supervision (equal); validation (equal); writing – original draft (lead).

## FUNDING INFORMATION

This research was supported by grants from the Ministry of Science and Technology, Taiwan (MOST 111‐2740‐B‐A49‐001 and 111‐2314‐B‐075‐043‐MY3); Taipei Veterans General Hospital (V106C‐162); the Department of Health, Taipei City Government (10701–62‐038, 10,901–62‐063).

## CONFLICT OF INTEREST STATEMENT

The authors declare no conflicts of interest.

## ETHICS STATEMENT

The study was approved by the Institutional Review Board of Taipei Veterans General Hospital. A written informed consent was signed by each participant.

## Supporting information


**Data S1:** Supplementary Information.

## Data Availability

The data that support the fundings of our study are available from the corresponding author upon reasonable request.
