# Supplementary material for: Decreased interleukin‐17RA expression is associated with good prognosis in patients with colorectal cancer and inhibits tumor growth and vascularity in mice
Source: Cancer Med. 2024 Mar 16;13(5):e7059. doi: 10.1002/cam4.7059 (PMC10943367; doi:10.1002/cam4.7059)

**Supplementary Material**

**Decreased interleukin-17RA expression is associated with good prognosis in patients with colorectal cancer and inhibits tumor growth and vascularity in mice**

**Jeng-Kai Jiang, Chi-Hung Lin, Ting-An Chang, Liang-Chuan Lo, Chien-Ping Lin, Ruey-Hwa Lu, Chih-Yung Yang**

**
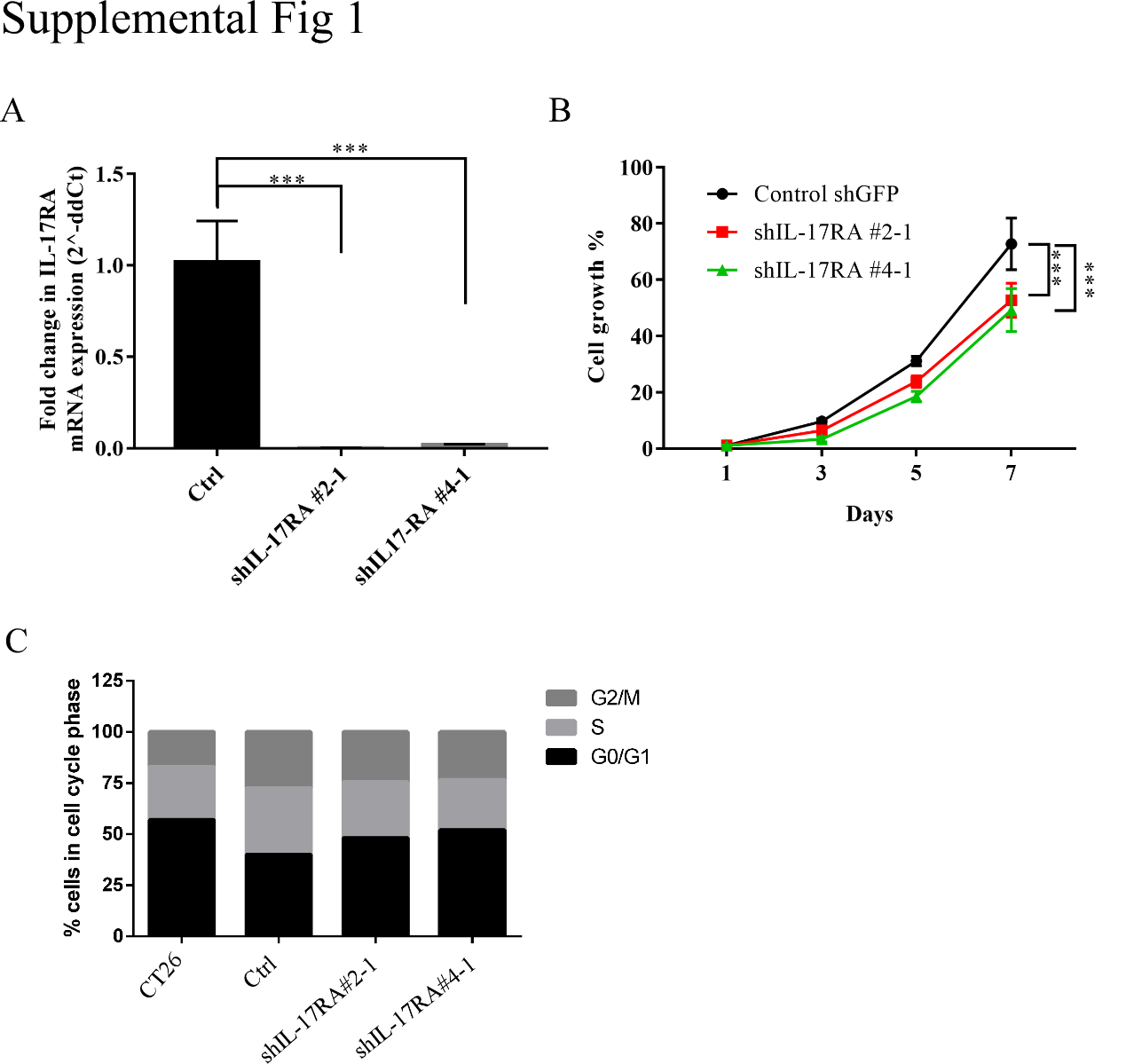
**

**Supplementary Figure 1:** Characterization of IL-17RA knockdown CT26 cells. (A) IL-17RA expression was examined using qRT-PCR in IL-17RA knockdown and control CT26 cells. (B) MTT assay showed that IL-17RA knockdown suppressed tumor cell growth in vitro. (C) IL-17RA knockdown had no significant effect on cell cycle compared to control CT26 cells.

**
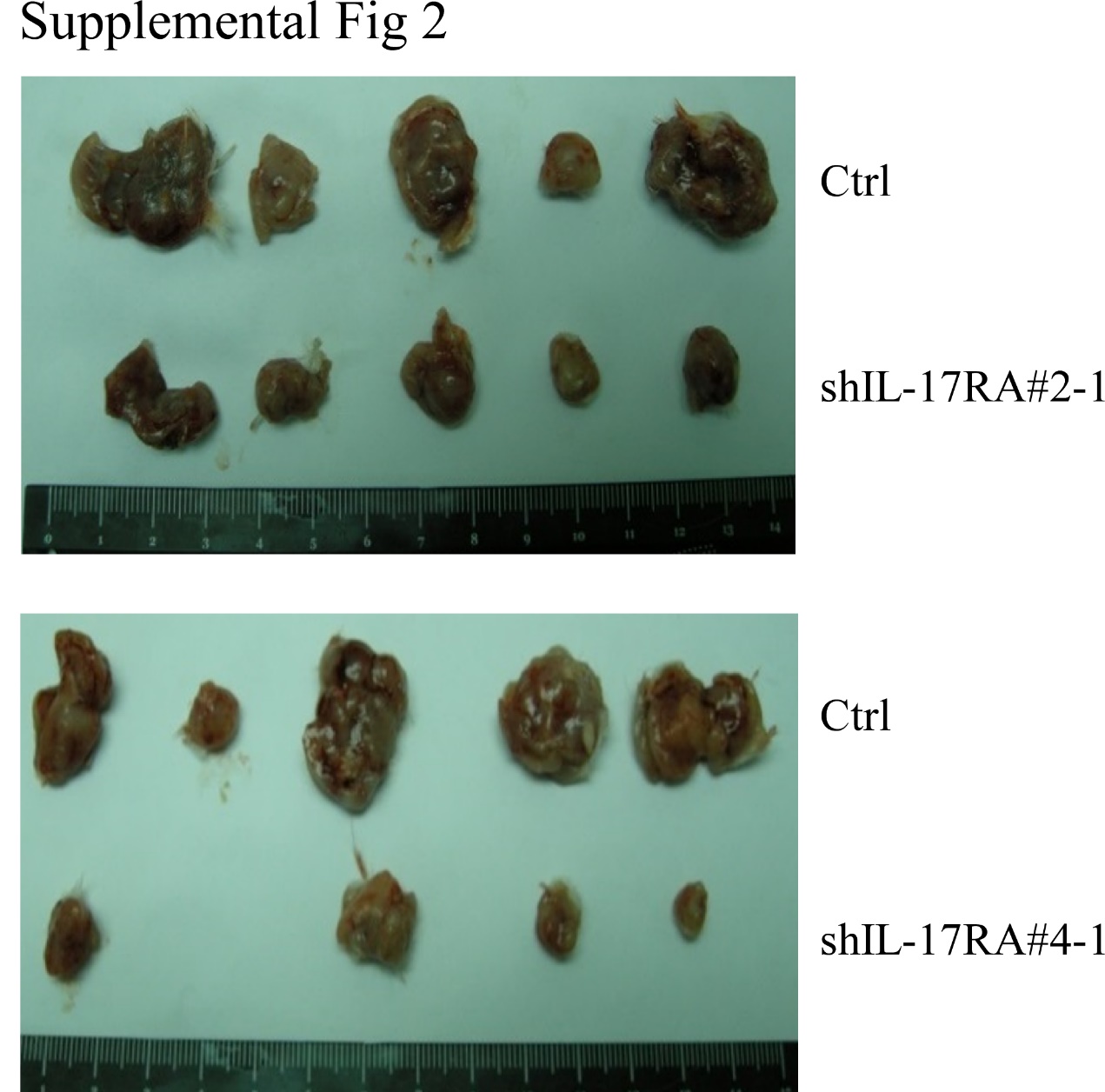
**

**Supplementary Figure 2:** Tumor masses from control shGFP tumors and shIL-17RA tumors.

Supplementary Table 1 The effect size and 95% confidence intervals in Figure 1

| Figure | Comparison | Mean of differences | 95% CI |
| --- | --- | --- | --- |
| 1B | Adjacent normal vs Tumor | -1.025 | -1.316 to -0.7337 |
| 1C | Adjacent normal vs Tumor | -0.4349 | -1.003 to -0.03058 |
| 1D | I vs IV | -0.6772 | -1.330 to -0.1655 |
|  | II vs IV | -0.8734 | -1.382 to -0.1911 |
|  |  | Hazard ratio | 95% CI |
| 1E | IL-17RA high vs low | 1.642 | 1.007 to 2.677 |
| 1F | IL-17RA high vs low | 2.255 | 1.286 to 3.954 |

Supplementary Table 2 The effect size and 95% confidence intervals in Figure 2

| Figure | Comparison | Mean of differences | 95% CI |
| --- | --- | --- | --- |
| 2A | 0 hr |  |  |
|  | Control shGFP vs. shIL-17RA #2-1 | 0.000 | -29.12 to 29.12 |
|  | Control shGFP vs. shIL-17RA #4-1 | 0.000 | -29.12 to 29.12 |
|  | 6 hr |  |  |
|  | Control shGFP vs. shIL-17RA #2-1 | 28.010 | -1.116 to 57.13 |
|  | Control shGFP vs. shIL-17RA #4-1 | 35.420 | 6.301 to 64.54 |
|  | 9 hr |  |  |
|  | Control shGFP vs. shIL-17RA #2-1 | 39.680 | 10.56 to 68.80 |
|  | Control shGFP vs. shIL-17RA #4-1 | 48.600 | 19.48 to 77.72 |
|  | 12 hr |  |  |
|  | Control shGFP vs. shIL-17RA #2-1 | 26.100 | -3.018 to 55.22 |
|  | Control shGFP vs. shIL-17RA #4-1 | 47.030 | 17.91 to 76.15 |
|  | 24 hr |  |  |
|  | Control shGFP vs. shIL-17RA #2-1 | 16.860 | -12.26 to 45.98 |
|  | Control shGFP vs. shIL-17RA #4-1 | 33.720 | 4.599 to 62.84 |
| 2B | Control shGFP vs. shIL-17RA #2-1 | 41.800 | 4.738 to 78.86 |
|  | Control shGFP vs. shIL-17RA #4-1 | 85.400 | 45.79 to 125.0 |
| 2C | Control shGFP vs. shIL-17RA #2-1 | 265.000 | 226.7 to 303.3 |
|  | Control shGFP vs. shIL-17RA #4-1 | 265.800 | 231.5 to 300.1 |

Supplementary Table 3 The effect size and 97.5 % confidence intervals in Figure 3

| Figure | Comparison | Mean of differences | 97.5% CI |
| --- | --- | --- | --- |
| 3A | E-cadherin |  |  |
|  | Control shGFP vs. shIL-17RA #2-1 | 0.11 | -1.321 to 1.541 |
|  | Control shGFP vs. shIL-17RA #4-1 | 0.3006 | -0.4528 to 1.054 |
|  | N-cadherin |  |  |
|  | Control shGFP vs. shIL-17RA #2-1 | 0.4638 | 0.07441 to 0.8532 |
|  | Control shGFP vs. shIL-17RA #4-1 | 0.4589 | 0.3374 to 0.5803 |
|  | Slug |  |  |
|  | Control shGFP vs. shIL-17RA #2-1 | 0.2746 | -0.3116 to 0.8609 |
|  | Control shGFP vs. shIL-17RA #4-1 | 0.4051 | -0.1392 to 0.9494 |
|  | Vimentin |  |  |
|  | Control shGFP vs. shIL-17RA #2-1 | 0.3653 | -0.1430 to 0.8737 |
|  | Control shGFP vs. shIL-17RA #4-1 | 0.4766 | 0.4001 to 0.5532 |
|  | IL-17RA |  |  |
|  | Control shGFP vs. shIL-17RA #2-1 | 0.6019 | 0.1927 to 1.011 |
|  | Control shGFP vs. shIL-17RA #4-1 | 0.72 | 0.6189 to 0.8211 |
| 3B | E-cadherin |  |  |
|  | Control shGFP vs. shIL-17RA #2-1 | 0.01615 | -0.2436 to 0.2759 |
|  | Control shGFP vs. shIL-17RA #4-1 | -0.1353 | -0.5061 to 0.2354 |
|  | N-cadherin |  |  |
|  | Control shGFP vs. shIL-17RA #2-1 | 0.2687 | 0.1416 to 0.3959 |
|  | Control shGFP vs. shIL-17RA #4-1 | 0.3926 | 0.05883 to 0.7263 |
|  | Slug |  |  |
|  | Control shGFP vs. shIL-17RA #2-1 | 0.324 | 0.04753 to 0.6004 |
|  | Control shGFP vs. shIL-17RA #4-1 | 0.458 | 0.1033 to 0.8126 |
|  | Vimentin |  |  |
|  | Control shGFP vs. shIL-17RA #2-1 | 0.8395 | 0.5027 to 1.176 |
|  | Control shGFP vs. shIL-17RA #4-1 | 0.7331 | 0.6529 to 0.8134 |
|  | IL-17RA |  |  |
|  | Control shGFP vs. shIL-17RA #2-1 | 0.2289 | 0.1757 to 0.2820 |
|  | Control shGFP vs. shIL-17RA #4-1 | 0.3014 | 0.04659 to 0.5563 |

Supplementary Table 4 The effect size and 95% confidence intervals in Figure 4

| Figure | Comparison | Mean of differences | 95% CI |
| --- | --- | --- | --- |
| 4A | Control shGFP vs. shIL-17RA #2-1 | 3.472 | 2.425 to 4.519 |
|  | Control shGFP vs. shIL-17RA #2-1 | 3.625 | 2.578 to 4.672 |
| 4B | Control shGFP vs. shIL-17RA | 0.1797 | 0.07224 to 0.2872 |
| 4C | Control shGFP vs. shIL-17RA | 2.9 | 1.600 to 3.900 |
| 4D | Control shGFP vs. shIL-17RA | -0.06857 | -0.2857 to 0.1486 |
| 4E | Control shGFP vs. shIL-17RA | 0.7211 | 0.1185 to 1.324 |
| 4F | Control shGFP vs. shIL-17RA | 0.455 | -0.03000 to 1.230 |

Supplementary Table 5 The effect size and 95% confidence intervals in Table 1

|  | IL-17RA high | IL-17RA low |  |  |  |
| --- | --- | --- | --- | --- | --- |
|  | N = 58 | N = 75 | Odds ratio | 95% CI | p |
| Age |  |  |  |  |  |
| ≥ 65 | 39 | 35 | 2.346 | 1.164 to 4.674 | 0.0223* |
| < 65 | 19 | 40 | 1 |  |  |
| Gender |  |  |  |  |  |
| Male | 36 | 47 | 0.9749 | 0.4806 to 1.977 | >0.9999 |
| Female | 22 | 28 | 1 |  |  |
| TNM Stage |  |  |  |  |  |
| IV | 23 | 14 | 1.125 | 0.3946 to 3.196 | >0.9999 |
| III | 15 | 19 | 1.776 | 0.6488 to 4.917 | 0.4221 |
| II | 12 | 24 | 3.696 | 1.229 to 9.839 | 0.0211* |
| I | 8 | 18 | 1 |  |  |
| T stage |  |  |  |  |  |
| T3-T4 | 51 | 51 | 3.429 | 1.357 to 8.664 | 0.0075** |
| T1-T2 | 7 | 24 | 1 |  |  |
| N stage |  |  |  |  |  |
| N2 | 19 | 14 | 2.714 | 1.206 to 6.518 | 0.0211* |
| N1 | 14 | 11 | 2.545 | 1.055 to 6.561 | 0.0587 |
| N0 | 25 | 50 | 1 |  |  |
| M stage |  |  |  |  |  |
| M1 | 23 | 14 | 2.863 | 1.308 to 6.269 | 0.0108* |
| M0 | 35 | 61 | 1 |  |  |
| Differentiation |  |  |  |  |  |
| Poor | 4 | 4 | Infinity | 1.000 to Infinity | 0.1429 |
| Moderate | 54 | 70 | Infinity | 0.6475 to Infinity | 0.261 |
| Well | 0 | 3 | 1 |  |  |
| Location |  |  |  |  |  |
| Right colon | 19 | 27 | 0.8042 | 0.3265 to 1.989 | 0.8131 |
| Left colon | 25 | 32 | 0.8929 | 0.3514 to 2.059 | 0.8242 |
| Rectal | 14 | 16 | 1 |  |  |
| CEA (5 ng/ml) |  |  |  |  |  |
| > 5 | 31 | 25 | 2.289 | 1.124 to 4.660 | 0.0318* |
| ≤ 5 | 26 | 48 | 1 |  |  |
| CA19-9 (U/ml) |  |  |  |  |  |
| > 37 | 18 | 14 | 1.876 | 0.8313 to 4.235 | 0.1504 |
| ≤37 | 37 | 54 | 1 |  |  |
| TIL |  |  |  |  |  |
| Positive | 14 | 16 | 1.173 | 0.5200 to 2.574 | 0.8346 |
| Negative | 44 | 59 | 1 |  |  |

Supplementary Table 6 The raw data of 4 independent experiments in Figure 3A


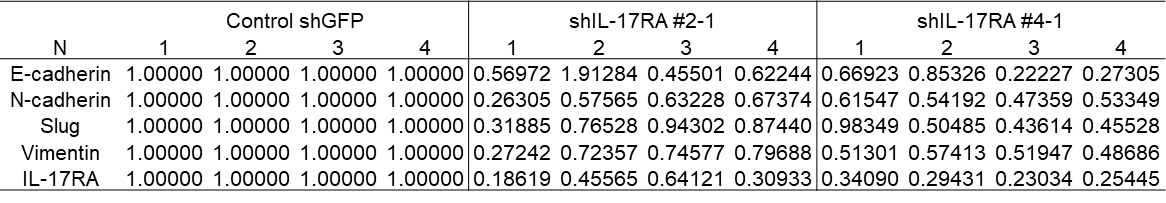

Supplement: Supplementary file 1 — Data S1: Supplementary Information. [file CAM4-13-e7059-s001.docx]
